# Melanotic Oncocytic Metaplasia of the Nasopharynx: An Unusual Case Report

**DOI:** 10.22038/ijorl.2021.51928.2759

**Published:** 2021-07

**Authors:** Barbara Verro, Enzo Chianetta, Giuseppe Greco, Carmelo Saraniti

**Affiliations:** 1 *Department of * *Biomedicine* *, Neurosciences and Advanced Diagnostic, University of Palermo, Palermo, Italy.*

**Keywords:** Metaplasia, Nasopharynx, Nasopharynx pathology, Rare diseases

## Abstract

**Introduction:**

Melanotic oncocytic metaplasia of the nasopharynx is an uncommon disease, usually asymptomatic, that could be misdiagnosed for melanoma, because of its macroscopic features. For this reason, is necessary to know it thoroughly and to take it into account in the differential diagnosis.

**Case Report:**

A 69-year-old Italian woman presented to our Otorhinolaryngology Clinic with a 1-month history of sore throat. She has been a smoker for several years. During the nasopharyngoscopic examination, grey-brown, irregular and slightly elevated lesions, measuring few millimetres, were found near the right Eustachian tube opening. The preliminary diagnostic hypothesis was malignant disease. After biopsy and histopathological assessment, the lesion was diagnosed as melanotic oncocytic metaplasia of the nasopharynx that is a benign and rare disease. So, given the multiple lesions and their benign nature, they were controlled with regular nasoscopic examinations.

**Conclusion:**

Melanotic oncocytic metaplasia is a benign lesion of the nasopharynx and it is necessary to emphasize the importance of its clinical awareness for differential diagnosis with malignant lesions.

## Introduction

Melanotic oncocytic metaplasia (MOM) of the nasopharynx is a rare lesion first described by Shek et al. in 1995 (1). To our knowledge, only 33 cases of MOM have been reported in literature. Macroscopically, MOM appears as simple or multiple small black or brown mucosal lesions, usually sited near the Eustachian tube opening (2-6). 

Moreover, it is often an incidental finding during endoscopic evaluation because related symptoms are uncommon (4). Although its macroscopic features could be mistaken for malignant tumour, as melanoma, MOM is a benign lesion. 

In this study, we present the first case of 69-year-old Caucasian (Italian) woman with incidental finding of MOM of the nasopharynx.

## Case Report

In 2019, a 69-year-old Italian woman presented to our ENT Clinic with a 1-month history of sore throat. She was a smoker with the consumption of 15 cigarettes per day for several years. As for the rest, her past medical history was unremarkable. 

**Fig 1:**
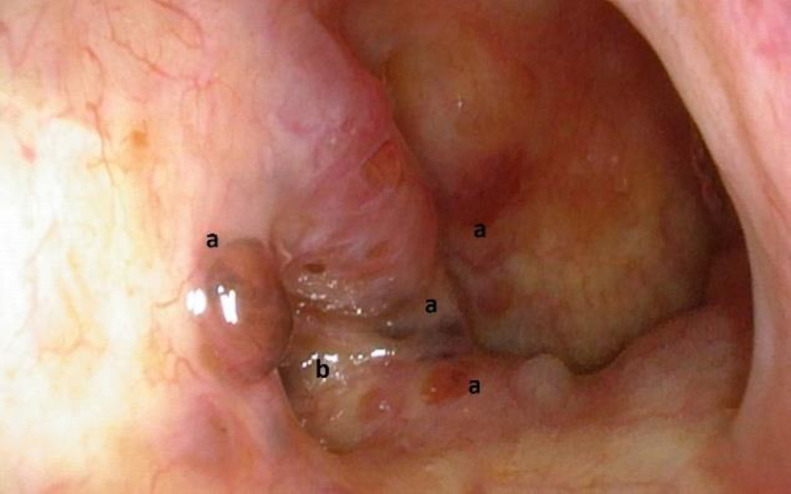
Melanotic oncocytic metaplasia of the nasopharynx. The figure shows multiple, grey-brown, irregular and slightly elevated nodules (a), with clearly identified margins, around the right Eustachian tube opening (b).

So, a nasopharyngoscopic examination was performed with the incidental finding of multiple, grey-brown, irregular and slightly elevated lesions, ranging from 2 to 4 mm in the maximal diameter, with clearly identified margins, near the right Eustachian tube opening (Fig.1). 

The overlying mucosa was intact, without signs of necrosis or bleeding when touched. Melanoma, melanosis or malignant tumour were suspected on macroscopic evaluation. Examination of neck, nasal cavity and larynx didn’t reveal abnormalities. Under local anaesthesia, one of the larger lesions was biopsied for histological assessment. The surgical specimen was a single, grey-brown, mucosal piece measuring 0.2x0.2x0.1 cm. Microscopic examination revealed a well demarcated lesion, covered by normal respiratory epithelium and composed by several seromucinous glands with diffuse oncocytic metaplasia. Moreover, scattered brown pigments was found in the cytoplasm of oncocytic cells. They were positive for Fontana-Masson staining and negative for Pears’s ones, confirming the melanin nature. There weren’t either cytological atypia nor mitotic figures in the epithelial cells of the glands. Immunohistochemically, dendritic cells were immunoreactive to S-100 protein and negative for HMB-45. Thus, based on these findings, the lesion was assessed as melanotic oncocytic metaplasia of the nasopharynx. Considering the benign nature of MOM, other similar lesions around the right Eustachian tube opening weren’t excised but only followed with periodic nasoscopic examinations. As previously stated, after diagnosis, the patient was controlled with nasopharynx endoscopy each three months. Thus, at nine-month follow-up, lesions in nasopharynx, near the right Eustachian tube opening, were unchanged, without progression to malignancy.

## Discussion

Melanotic oncocytic metaplasia (MOM) of the nasopharynx is an uncommon and little-known disease. Indeed, oncocytic metaplasia is most frequently detected in other sites, as salivary glands, thyroid and parathyroid glands (1,7). Histologically, MOM appears as oncocytic epithelial cells with intra-cytoplasmatic melanin pigments. The origin of this pigment is still unknown. According to the most shared hypothesis, the pigment is produced by melanocytes and transferred to adjacent oncocytic cells by their dendritic processes. Indeed, as reported in literature (8), stroma and epithelium of the nasal cavity present dendritic melanocytes. This hypothesis is confirmed by electron microscopy that doesn’t identify premature m

elanosome in oncocytic cells and by immunohistochemistry that finds S-100 positive and HMB-45 negative dendritic cells (5,9,10,11).

This metaplasia is often an incidental finding during nasopharyngeal examination for other disease since it’s usually asymptomatic, as well as in our case. However, given its siting near the Eustachian tube opening, the most common related symptoms are otitis media, tinnitus, nasal obstruction and rhinorrhea. Moreover, analysing the literature (Table.1), MOM usually occurs as multiple lesions with unilateral involvement, just as our patient who presented several small lesions around the right Eustachian tube opening.

**Table 1:** Review of literature about melanotic oncocytic metaplasia of nasopharynx

**Case**	**Author**	**Age**	**Sex**	**Uni/bilateral involvement**	**Number**	**Smoking history**
1	Shek (1)	67	M	Unilateral	Simple	Unknown
2		63	M	Unilateral	Simple	Unknown
3	Hirakawa (5)	64	M	Bilateral	Multiple	Unknown
4	Xue (12)	70	M	Unilateral	Multiple	None
5	Takano (2)	62	M	Unilateral	Multiple	Unknown
6	Sakaki (9)	80	M	Unilateral	Multiple	Yes
7		69	M	Unilateral	Simple	Yes
8		74	M	Unilateral	Simple	Unknown
9		74	F	Unilateral	Multiple	Unknown
10		68	M	Unilateral	Simple	Unknown
11		56	M	Unilateral	Simple	Unknown
12		63	M	Unilateral	Simple	Unknown
13	Li (13)	58	M	Bilateral	Multiple	Unknown
14	Kondo (3)	73	M	Bilateral	Multiple	None
15	Na (11)	72	M	Bilateral	Multiple	Yes
16		71	M	Unilateral	Multiple	Yes
17		51	M	Unilateral	Multiple	Unknown
18	Chang (10)	63	M	Unilateral	Multiple	Yes
19	Tajima (4)	57	M	Unilateral	Multiple	Yes
20	Lin (14)	60	M	Unilateral	Simple	Unknown
21	Uehara (8)	70	F	Bilateral	Multiple	Yes
22		61	F	Unilateral	Simple	Yes
23		74	M	Unilateral	Multiple	Yes
24	Li (15)	57	M	Unilateral	Simple	Yes
25		61	M	Unilateral	Simple	Yes
26		69	M	Unilateral	Multiple	Yes
27		56	M	Unilateral	Simple	Yes
28		58	M	Unilateral	Simple	Yes
29		52	M	Unilateral	Multiple	Yes
30		77	F	Unilateral	Simple	Yes
31		59	M	Unilateral	Simple	Yes
32		59	M	Unilateral	Simple	Yes
33	Chen (16)	75	M	Unilateral	Multiple	Yes
34	Present case	69	F	Unilateral	Multiple	Yes

Epidemiologically, most of the patients are men (8 men: 1 women) with a mean age of 70 years (range, 51 to 80 years). Moreover, to date, the literature reports only cases of Asiatic people affected by MOM. Hence, our case report differs from these since the patient is a 69-year-old Italian woman, thus the first case of European patient affected by MOM, to the best of our knowledge. 

Furthermore, in 20 of 33 cases of MOM reported in literature patients were smokers (3,4,7,8,15,16), as well as our patient who smokes about 15 cigarettes a day. Therefore, many authors suggested a correlation between smoking and MOM (9). This hypothesis is supported by male predominance of disease, given that the greater part of smokers is male. Moreover, since the finding of oncocytic changes increases with age and it’s considered a form of cellular degeneration (3,17), MOM may represent an age-related phenomenon, confirmed by its occurrence more frequent in elderly. However, the pathogenesis of MOM is still unknown. Someone postulated the role of nasopharynx bacteria in its pathogenesis (15), whereas others suggested a genetic predisposition given the frequent occurrence in Asia or a relation to Warthin tumour for the similar histological features and the association with smoking (16). Despite the lack of knowledge about its origin and causes, MOM can be considered a benign lesion. In literature, we didn’t find cases of recurrence or progression to malignancy. Thus, excision if it’s a single lesion or follow-up if they are multiple represent the proper management of MOM. 

## Conclusion 

Melanotic oncocytic metaplasia of nasopharynx is a rare disease and it is crucial to investigate it further because, although rare, it may be misdiagnosed as a malign and more serious lesion (i.e. melanoma) which would require a more invasive treatment and would be correlated to a worse prognosis. 

## Learning Points

- Melanotic oncocytic metaplasia of the nasopharynx is a rare and benign disease.
- This disease should be considered in differential diagnosis of small, grey-brown, lesions around the Eustachian tube opening (i.e. melanoma).
- Given the benign nature of this disease, excision if it’s a single lesion or follow-up if they are multiple represent the proper management.
